# Changes in Morphology, Gene Expression and Protein Content in Chondrocytes Cultured on a Random Positioning Machine

**DOI:** 10.1371/journal.pone.0079057

**Published:** 2013-11-11

**Authors:** Ganna Aleshcheva, Jayashree Sahana, Xiao Ma, Jens Hauslage, Ruth Hemmersbach, Marcel Egli, Manfred Infanger, Johann Bauer, Daniela Grimm

**Affiliations:** 1 Clinic for Plastic, Aesthetic and Hand Surgery, Otto-von Guericke-University, Magdeburg, Germany; 2 Institute of Biomedicine, Pharmacology, Aarhus University, Aarhus C, Denmark; 3 Deutsches Zentrum für Luft- und Raumfahrt, Biomedizinisches Wissenschafts- Unterstützungszentrum, Gravitationsbiologie, Köln, Germany; 4 Aerospace Biomedical Science & Technology, Space Biology Group, Lucerne School of Engineering and Architecture, Hergiswil, Switzerland; 5 Max-Planck-Institute for Biochemistry, Martinsried, Germany; Ohio State University, United States of America

## Abstract

Tissue engineering of chondrocytes on a Random Positioning Machine (RPM) is a new strategy for cartilage regeneration. Using a three-dimensional RPM, a device designed to simulate microgravity on Earth, we investigated the early effects of RPM exposure on human chondrocytes of six different donors after 30 min, 2 h, 4 h, 16 h, and 24 h and compared the results with the corresponding static controls cultured under normal gravity conditions. As little as 30 min of RPM exposure resulted in increased expression of several genes responsible for cell motility, structure and integrity (beta-actin); control of cell growth, cell proliferation, cell differentiation and apoptosis (TGF-β_1_, osteopontin); and cytoskeletal components such as microtubules (beta-tubulin) and intermediate filaments (vimentin). After 4 hours of RPM exposure disruptions in the vimentin network were detected. These changes were less dramatic after 16 hours on the RPM, when human chondrocytes appeared to reorganize their cytoskeleton. However, the gene expression and protein content of TGF-β_1_ was enhanced during RPM culture for 24 h. Taking these results together, we suggest that chondrocytes exposed to the RPM seem to change their extracellular matrix production behaviour while they rearrange their cytoskeletal proteins prior to forming three-dimensional aggregates.

## Introduction

Chondrocytes are the only mature cell type found in healthy human cartilage. Originating from chondroblasts and located in cartilaginous tissue, they produce an extracellular matrix consisting primarily of collagen type II, which is responsible for the structure, and of cartilage-specific proteoglycans (aggrecan), making up 5% to 10% of the wet weight of the cartilage [1], [2]. With an advanced Golgi apparatus and plenty of rough endoplasmic reticulum, chondrocytes are scattered in cartilage cavities, also called lacunae [2]. The blood supply of chondrocytes is facilitated through the perichondrium and synovial fluid. Embedded in cartilage, chondrocytes are not capable of cell division *in vivo* [1]. Loss (degradation) of cartilage leads to diseases such as osteoarthritis, which frequently occurs in elderly or overweight people and is characterized by progressive destruction of articular cartilage as well as remodeling of the periarticular bone and inflammation of the synovial membrane [1], [3], [4].

Autologous chondrocyte transplantation (ACT) is currently used to repair large joint defects with reasonable clinical outcome as well as improve the histology of the repair tissue [5]. However, in addition to the tremendous shortage of tissue and organ transplants, the yield of chondrocytes that can be harvested from small biopsies is very limited [5]. To obtain the required amounts of cells needed for the repair of a defect, chondrocytes must undergo several population doublings *in vitro*, which unfortunately correlates with a dramatic loss of the chondrogenic phenotype [5]. Moreover, sequelae are the major disadvantage of transplantations. As a consequence, methods to seed chondrocytes on scaffolds fixed into the defect site by arthroscopic or minimally invasive techniques are currently under investigation and use [6]. The scaffolds produced from either natural materials or synthetic polymers should be able to provide not only physical support for the cells, but also the chemical and biological cues needed to form functional tissues [6], [7]. The materials and their degradation products must be non-toxic and non-immunogenic and their degradation rate should match the rate of new tissue formation [7], [8]. But to date, these hurdles remain difficult to overcome.

Therefore, scaffold-free methods of cartilage repair are of great interest. An alternative tissue source could be cartilage engineered *in vitro* and subsequently implanted. Microgravity provides the possibility of creating three-dimensional differentiated tissue-like cell assemblies and offers research opportunities that may lead to the generation of replacement organs for transplantation [9]. Therefore, culturing of chondrocytes with the aim of engineering cartilage tissue, either *in vivo* at the site of damage or *in vitro* for subsequent implantation, is a cutting edge field to explore [10].

Researchers have found that microgravity offers many advantages in the area of tissue engineering, especially in promoting scaffold-free three-dimensional growth [10], [11]. Three-dimensional growth is accomplished because the cells are not driven against a solid surface and do not grow across a solid–liquid interface, as they do at the bottom of a Petri dish or a culture flask on Earth. However, the incubation of cells under microgravity (real or simulated) has a great impact on their growth and physiology [9]. We detected a number of molecular changes when we studied spheroid formation by follicular thyroid cancer cells cultured on a Random Positioning Machine (RPM), a device developed to achieve simulated microgravity [12]. Similar alterations were seen when adherent human endothelial EA.hy926 cells formed elongated or tube-like structures cultured on a RPM [13]. Several studies on chondrocytes, cultured either in Space (real microgravity) or on a ground-based facility for simulation of microgravity, revealed the formation of aggregates resembling cartilage. Chondrocytes are cells that are highly resistant to stress induced by altered gravity [10], [14]. Another publication proposed that the RPM may be a useful tool to produce cartilaginous tissue grafts with fewer cells [15]. We demonstrated that human chondrocytes, incubated for 18 days on a RPM, formed three-dimensional aggregates that may have developed from small spheroids detected after 7 days [10]. However, the early changes in chondrocytes under microgravity conditions remain unclear.

The principal aim of this study was to investigate the early cellular events that occurred in hip joint chondrocytes from six different donors, cultured on a RPM to initiate the formation of three-dimensional aggregates. For this purpose, we investigated changes in the morphology of the cytoskeleton, which contains three distinct filamentous biopolymers (microtubules, microfilaments, and intermediate filaments) in vertebrates. We focused on gene expression and protein content after 30 min, 2 hours, 4 hours, 16 hours and 24 hours of incubation on the RPM and compared the results with the corresponding static controls, cultured under normal gravity conditions (1 *g*).

## Materials and Methods

### Ethics Statement

The biopsies for the chondrocyte establishment were collected after obtaining the patients’ written consent. The protocol had been approved by the local ethics committee under the Danish National Committee on Research Ethics [16].

### Establishment of Different Primary Chondrocyte Cell Cultures

Six primary cell cultures of human chondrocytes derived from hip joint cartilage were used for this study. Four of them were purchased from Provitro**®** (Berlin, Germany). Two further chondrocyte cell cultures were kindly provided by the Orthopaedic Research Lab, Aarhus University Hospital, and Interdisciplinary Nanoscience Centre (iNANO), Aarhus University, Aarhus C, Denmark. These primary cells were isolated from cartilage biopsies according to a protocol described by Foldager et al. [16]. The biopsies have been collected from the intercondylar groove in the distal femur from healthy patients undergoing anterior cruciate ligament reconstruction after obtaining the patients’ written consent.

All cultures were grown in DMEM/F-12 medium supplemented with 10% fetal calf serum (Provitro**®**, Berlin, Germany), 100 IU penicillin/mL and 100 µg streptomycin/mL (Biochrom**®**, Berlin, Germany), in 75 cm^2^ cell culture flasks (Sarstedt**®**, Nümbrecht, Germany). Air exchange in the culture flasks was assured using a waterproof, but air-permeable membrane in the cap of the flasks.

Cells cultured in our laboratory from frozen stocks were utilized at passage levels three to eight for all tests. Fresh human chondrocytes were first expanded in a monolayer for 7–10 days to reach confluence. Sub-confluent monolayers (1×10^6^ cells/cm^2^) were randomized to the following study groups: 300 static control cultures for the PCR analyses (n = 10 for each time point and donor), 300 static control cultures for the Western Blot analyses (n = 10 for each time point and donor), 300 samples for the RPM experiments for the PCR analyses (n = 10 for each time point and donor) and 300 samples for the RPM experiments for Western Blot analyses (n = 10 for each time point and donor). In addition, cells of each chondrocyte cell line were seeded in Super Cell chamber slides (1 *g* and RPM group) for histochemical and immunocytochemical staining.

### Random Positioning Machine

**Figure 1:**
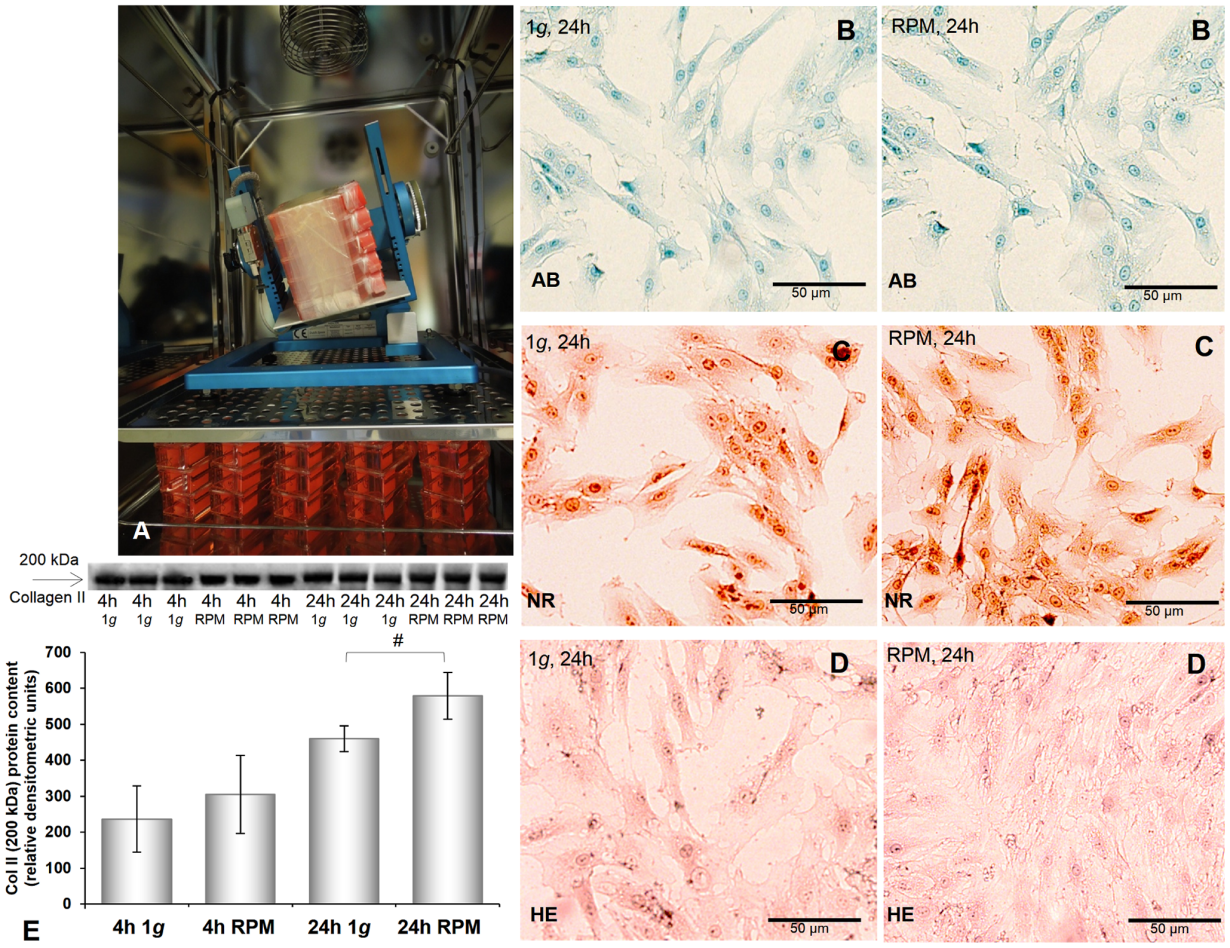
Culture flasks containing sub-confluent chondrocyte monolayers, histological evaluation, and Western blot analysis of type 2 collagen of chondrocytes cultured on the RPM or under normal gravity (1 *g*). **A:** Culture flasks containing sub-confluent chondrocyte monolayers were completely filled with medium and fixed on the RPM, as close as possible to the centre of the platform. Static 1 *g* control cultures were placed in the same incubator. Alcian Blue staining (AB; **B**) indicates production of proteoglycans. Neutral red staining (NR; **C**) depicts the viability of the chondrocyte cells. Haematoxylin-eosin staining (HE; **D**) gives an overview of tissue structure and cell distribution. **E:** Western blot analyses of type 2 collagen. Type 2 collagen is significantly elevated in 24 h RPM samples compared to corresponding 1 *g* cultures. Data are given as mean ± standard deviation; *P<0.005 vs. corresponding 1 *g.*

Microgravity conditions were simulated using a Desktop Random Positioning Machine (RPM), manufactured by Dutch Space (an Astrium Company, Leiden, Netherlands) [17]. The RPM is a laboratory instrument, enabling the position of an accommodated (biological) experiment in three-dimensional space to be randomly altered by dedicated software running on a personal computer. Culture flasks containing sub-confluent monolayers were completely filled with medium devoid of air bubbles and fixed on the RPM, as close as possible to the centre of the platform (Fig. 1), which was then rotated at a speed of 60°/s using the real random mode (random speed and random direction) of the machine. The RPM was positioned in a commercially available incubator set at 37°C and supplying 5% CO_2_. 1 *g* ground control cultures, treated in parallel in identical equipment, were placed in the same incubator as the RPM (Fig. 1).

### Histological Analysis and Viability Staining

Following the RPM run, the chondrocytes were stained with haematoxylin and eosin to gain an overview of the tissue structure and cell distributions [10]. The viability and the deposition of glycosaminoglycans of the control and RPM-exposed cells were examined using Neutral Red and Alcian Blue staining, respectively (both from Sigma, Taufkirchen, Germany). For this purpose, the cells were fixed for 10 min with 4% paraformaldehyde (PFA in DPBS), washed twice with DPBS, stained with a drop of the diluted staining solution (diluted according to the manufacturer’s instructions), washed twice with DPBS again and mounted with Entellan® (Merck, Darmstadt, Germany) for further microscopic analysis.

### Immunofluorescence

For immunofluorescence staining, the cells (1×10^6^ cells/cm^2^) were seeded into several four-chamber Super Cell chamber slides (BD, Heidelberg, Germany) and placed in the incubator (37°C, 5% CO_2_) overnight, until they attached to the slides. The next day, the slides were completely filled with medium, sealed with paraffin, and placed on the RPM for the run. After the run, the chondrocytes were washed twice with DPBS, fixed for 30 min with 4% paraformaldehyde (4°C), and permeabilised with Triton X-100 (Sigma, Taufkirchen, Germany). The cells were then washed twice in DPBS and incubated with primary antibody for 24 h at room temperature. The morphology of the microtubules (beta-tubulin, 1∶1000; Cell Signaling Technology, Inc., Danvers, MA, USA), and intermediate filaments (vimentin, 1∶1000; Cell Signaling Technology, Inc., Danvers, MA, USA) was determined by indirect immunofluorescence (IIF). In addition, BMP-2 (dilution 1∶100; Abcam, Cambridge, UK) was evaluated by IIF. After incubation with primary antibody, the chondrocytes were washed twice with DPBS and incubated for 2 h with the secondary FITC-tagged antibody, used at a dilution of 1∶500 (Cell Signaling Technology, Inc., Danvers, MA, USA). For nuclear staining, the cells were further incubated with propidium iodide (1∶100; Invitrogen, Carlsbad, CA, USA) for 10 min, mounted with Vectashield® immunofluorescence mounting medium (Vector, Burlingame, CA, USA), and analysed microscopically.

### F-actin Staining

F-actin was visualised by means of rhodamine-phalloidin staining (Molecular Probes**®**, Eugene, OR, USA) [13], [18]. For this purpose, seeded cells were fixed for 30 min with 4% PFA (in DPBS), washed twice with DPBS, incubated with 5 µg/ml fluorescent phalloidin conjugate solution in PBS/1% BSA for at least 20 min at room temperature and then washed several times with PBS to remove unbound phalloidin conjugate. Afterwards, the nuclei were stained with Hoechst 33342 (Molecular Probes**®**, Eugene, OR, USA) for 5 min and washed twice with DPBS. For evaluation, the samples were mounted with Vectashield® (Vector, Burlingame, CA, USA) and analysed microscopically.

### Microscopy

The viability and morphology of the cells grown on four-chamber Super Cell slides (BD, Heidelberg, Germany) were examined by phase-contrast microscopy (Olympus, Hamburg, Germany) immediately after RPM exposure. Immunofluorescence and F-actin staining were analysed with a Zeiss 510 META inverted confocal laser scanning microscope (Zeiss, Germany), equipped with a Plan-Apochromat 63×1.4 objective. Excitation and emission wavelengths were: **λ**_exc_ = 488 nm and **λ**_em_ = ≥505 nm for FITC. All samples were analysed with the help of the image analysis program Scion Image (Version 1.63 MacOs, Scion Corporation, USA).

### RNA Isolation

Ten cell culture flasks from each time point and donor were used for RNA extraction. The cells were scraped off using cell scrapers (Sarstedt, Nümbrecht, Germany), transferred to 50 ml tubes, and pelleted by centrifugation (2500 g, 10 min, 4°C). The RNeasy Mini Kit (Qiagen, Hilden, Germany) was used according to the manufacturer’s instructions to isolate total RNA. RNA concentrations and quality were determined spectrophotometrically at 260 nm using a NanoDrop instrument (Thermo Scientific, Wilmington, DE, USA). The isolated RNA had an A260/280 ratio of 1.5 or higher. cDNA designated for quantitative real-time PCR was then obtained using the First-Strand cDNA Synthesis Kit (Fermentas, St. Leon-Rot, Germany) using 1 µg of total RNA in a 20-µl reaction mixture at room temperature. Details of this method are available elsewhere [11], [19], [20].

### Quantitative Real-time PCR

Quantitative real-time PCR [11], [19], [20] was used to determine the expression levels of the genes of interest (Table 1): *ACTA2*, *ACTB*, *VIM*, *TUBB6*, *ITGB1*, *BMP2*, *OPN*, and *TGFB1* genes after 30 min, 2 h, 4 h, 16 h, and 24 h incubation under simulated microgravity (µg) compared to the static control group (1 *g*). Primer Express**®** software (Applied Biosystems, Darmstadt, Germany) was employed to design appropriate primers with a T_m_ of about 60°C (Table 1). The primers were synthesised by TIB Molbiol (Berlin, Germany). All assays were run on a 7500 fast real-time PCR system using the Fast SYBR**®** Green PCR Master Mix (Applied Biosystems, Darmstadt, Germany). The reaction volume was 25 µL including 1 µL of template cDNA and a final random hexamer primer concentration of 500 nM. PCR conditions were as follows: 20 s at 95°C, 40 cycles of 3 s at 95°C, and 30 s at 60°C, followed by a melting curve analysis step (temperature gradient from 60 to 95°C with +0.3°C/cycle).

**Table 1:** Primers used for quantitative real-time PCR.

Gene	Primer name	Sequence (5′→3′)
**18S rRNA**	18S-F	GGAGCCTGCGGCTTAATTT
	18S-R	CAACTAAGAACGGCCATGCA
**ACTA2**	ACTA2-F	GAGCGTGGCTATTCCTTCGT
	ACTA2-R	TTCAAAGTCCAGAGCTACATAACACAGT
**ACTB**	ACTB-F	TGCCGACAGGATGCAGAAG
	ACTB-R	GCCGATCCACACGGCGTACT
**BMP2**	BMP2-F	GACCTGTATCGCAGGCACTCA
	BMP2-R	TCGTTTCTGGTAGTTCTTCCAAAGA
**INTB1**	INTB1-F	GAAAACAGCGCATATCTGGAAATT
	INTB1-R	CAGCCAATCAGTGATCCACAA
**KRT8**	KRT8-F	GATCTCTGAGATGAACCGGAACA
	KRT8-R	GCTCGGCATCTGCAATGG
**OPN**	OPN-F	CGAGGTGATAGTGTGGTTTATGGA
	OPN-R	CGTCTGTAGCATCAGGGTACTG
**TGFB1**	TGFB1-F	CACCCGCGTGCTAATGGT
	TGFB1-R	AGAGCAACACGGGTTCAGGTA
**TUBB6**	TUBB6-F	GTGCGGTCTGGGCCTTTT
	TUBB6-R	CTCCGTGTAGTGCCCTTTCG
**VIM**	VIM-F	TTCAGAGAGAGGAAGCCGAAAAC
	VIM-R	AGATTCCACTTTGCGTTCAAGGT

All sequences are given in 5′–3′ direction.

If all amplicons showed a single Tm similar to the one predicted by the Primer Express**®** software, the PCR reactions were considered specific. Every sample was measured in triplicate. The comparative C_T_ (**ΔΔ**C_T_) method was used for the relative quantification of transcription levels. 18S rRNA was used as a housekeeping gene to normalise expression data.

### Western Blot Analysis

Gel electrophoresis, trans-blotting, and densitometry were carried out following routine protocols as described previously [21]–[23]. An equal amount of 20 µL of lysate, containing 3 µg/µL protein, was loaded onto SDS-PAGE. Each Western blot was performed three times for each donor, whereby each sample was applied three times. Anti-beta-actin, anti-beta-tubulin, anti-vimentin, and anti-TGF-β_1_ antibodies were used at a dilution of 1∶1000 (Cell Signaling Technology, Inc., Danvers, MA, USA); anti-osteopontin antibody was used at a dilution of 1∶1000 (Rockland Immunochemicals Inc., Gilbertsville, USA), as well as anti-integrin-beta_1_ antibody (Epitomics, Burlingame, USA); anti-BMP-2 antibody was applied at a dilution of 1∶100 (Life Technologies, Darmstadt, Germany). The secondary, HRP-linked antibody was utilised at a dilution of 1∶3000 (Cell Signaling Technology, Inc., Danvers, MA, USA). Protein from the 3T3 Swiss Albino cell line (ATCC**®**) was used as a positive control [24].

For the densitometric quantification of the bands, the membranes were analysed using ImageJ software (U.S. National Institutes of Health, Bethesda, MD, USA; http://rsb.info.nih.gov/ij/).

### Statistical Analysis

All statistical analyses were performed using SPSS 21.0 (SPSS, Inc., Chicago, IL, USA, 2012). We tested all parameters achieved via PCR and Western blot analyses using one-way ANOVA or the Mann-Whitney U test (depending on the results of a normality test). All data are expressed as means ± standard deviation (SD). Differences were considered significant at p<0.05 (#).

## Results

### Morphological Changes

In order to investigate how the morphology and growth behaviour of human chondrocytes are altered by the RPM (Fig. 1), we performed several experiments for 30 min, 2 h, 4 h, 16 h, and 24 h, during which the chondrocytes were incubated on the RPM or under normal static gravity conditions (1 *g*).

After 24 h of incubation, the chondrocytes cultured either on the RPM or at 1 *g* showed equal viability. As determined using Neutral Red staining, all chondrocyte cultures remained alive and healthy (Fig. 1). This was confirmed by Hoechst 33342 staining of the chondrocytes (see below).

Moreover, the cells showed no differences in cell morphology (Fig. 1). However, the deposition of acidic polysaccharides such as glycosaminoglycans, as illustrated by Alcian Blue (AB) staining, was reduced after 24 h incubation on the RPM compared to normal 1 *g* samples: The intensity of the staining decreased especially around the nuclei (Fig. 1). Western blot analyses of collagen type 2 revealed high levels of this extracellular matrix protein in all chondrocyte samples after 4 h and 24 h (Fig. 1). The amount of collagen type 2 increased over time and this increase was significant after 24 h RPM exposure.

### RPM Exposure Induces Early Effects on the Cytoskeleton and Changes BMP-2 Expression

Previous studies have shown that changes in growth behaviour are often accompanied by alterations in the cytoskeleton [12], [18]. Therefore, we performed immunofluorescence staining of several cytoskeletal components. The cells were treated with antibodies against β-tubulin, vimentin, and bone morphogenetic protein 2 (BMP-2), which plays an important role in the development of cartilage. The bound antibodies were visualised according to the technique described above. The results were compared with the corresponding 1 *g* cells.

**Figure 2:**
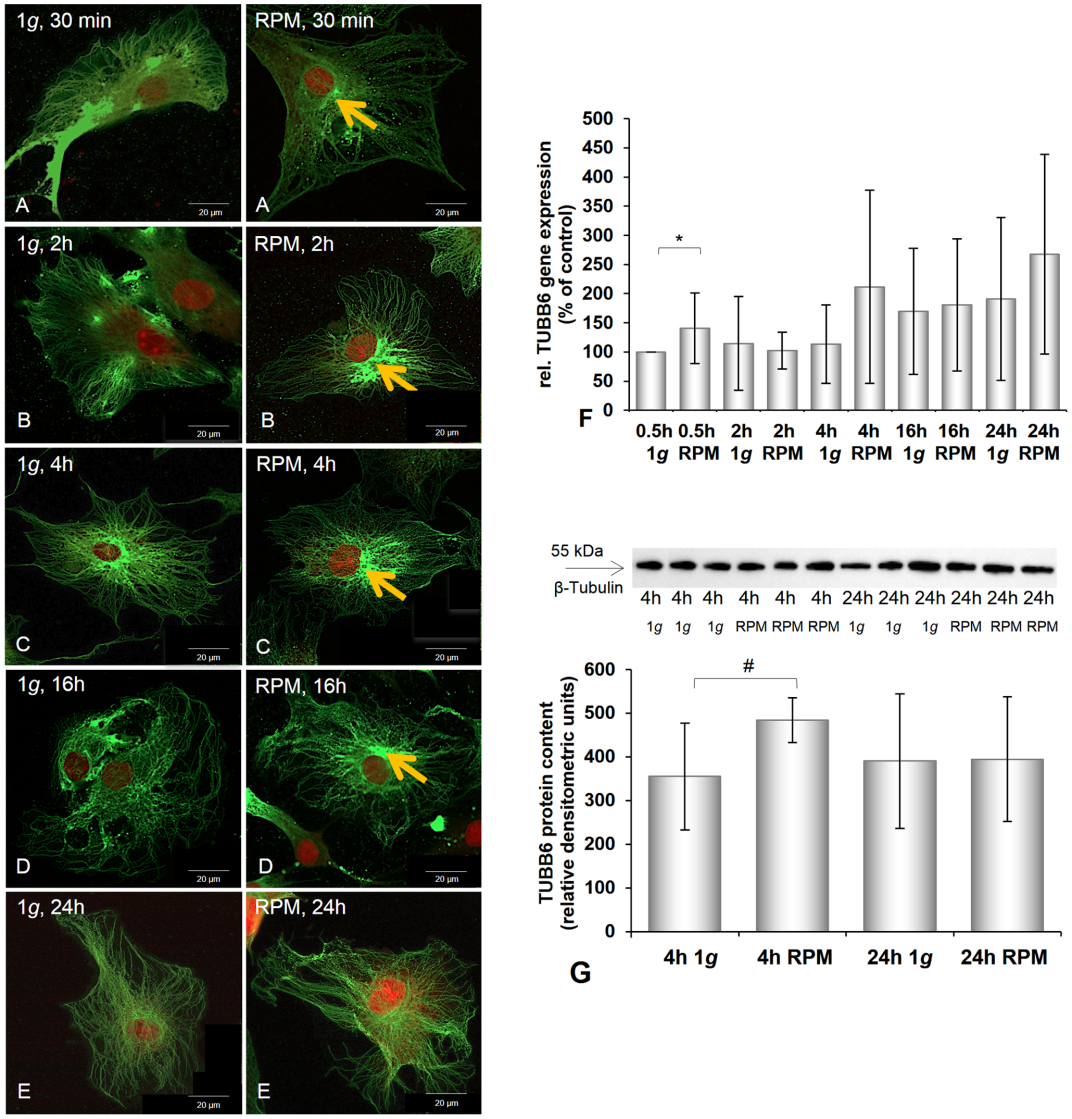
Immunofluorescence, gene expression and Western blot analysis of beta-tubulin of chondrocytes cultured at 1 *g* or on the RPM. **A–E:** Beta-tubulin immunofluorescence of chondrocytes cultured at 1 *g* or on the RPM for different times (30 min (**A**), 2 h (**B**), 4 h (**C**), 16 h (**D**), 24 h (**E**)). Control cells showed a similar distribution of β-tubulin, while chondrocytes exposed to the RPM exhibited a perinuclear accumulation of β-tubulin (orange arrows). **F:** Gene expression of *TUBB6* was significantly elevated after 0.5 h, but did not change significantly during the rest of the 24 h of culturing on the RPM. **G:** Western blot analysis of ß-tubulin protein: The protein content was significantly elevated after 4 h RPM exposure. After 24 h no change was detectable. Data are given as mean ± standard deviation; #P<0.05; *P<0.005.

**Figure 3:**
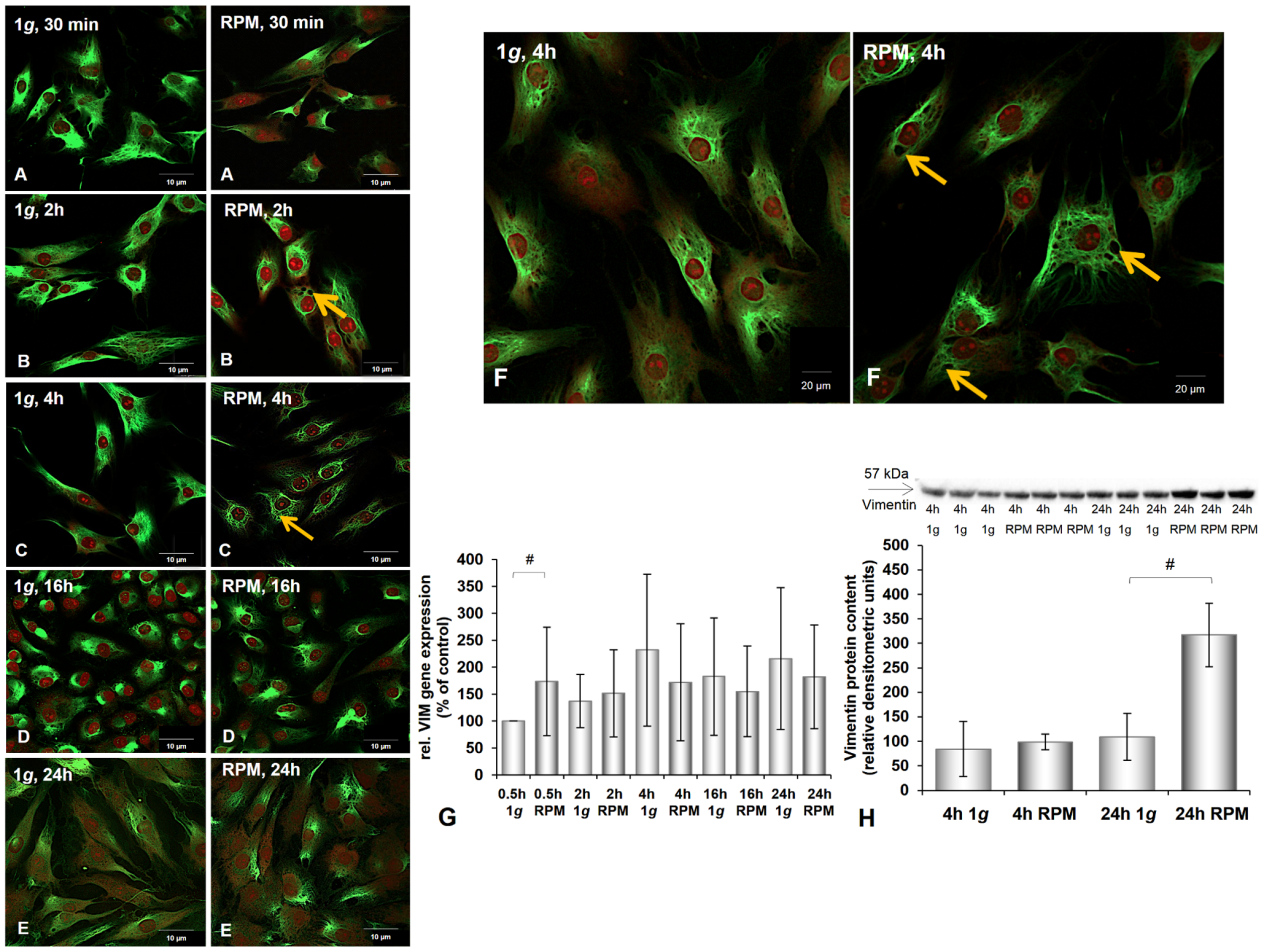
Immunofluorescence, gene expression, and Western blot analysis of vimentin of chondrocytes cultured at 1 *g* or on the RPM. **A–F:** Vimentin immunofluorescence of chondrocytes cultured at 1 *g* or on the RPM for different times (30 min (**A**), 2 h (**B**), 4 h (**C**), 16 h (**D**), 24 h (**E**)). When chondrocytes were exposed to the RPM for at least 2 h, vimentin staining accumulated around the nucleus and small holes (orange arrows) were detected in the outer cellular membrane, whereas a rather even vimentin distribution was seen in chondrocytes cultured under 1 *g* control conditions**. F:** Vimentin cytoskeleton after 4 h incubation of chondrocytes on the RPM and at 1 *g*. Orange arrows indicate small holes in the cytoplasm occurring after 4 h. **G:** *VIM* gene expression: the gene was significantly up-regulated after 0.5 h in chondrocytes cultured on the RPM compared with the corresponding control. **H:** Western blot analysis of vimentin: The amount of vimentin protein increased significantly after 24 h. Data are given as mean ± standard deviation; #P<0.05; *P<0.005.

Our data clearly show that after incubation on the RPM lasting 30 min, the cytoskeleton had disintegrated. Several alterations in various components of the cytoskeleton were observed. We detected changes in the microtubules (Fig. 2) and in the intermediate filaments (Fig. 3): The cells exhibited a perinuclear accumulation of beta-tubulin after 30 min of RPM exposure (Fig. 2), while beta-tubulin was distributed across the whole body of the chondrocytes as long as the cells grew under normal 1 *g* conditions (Fig. 2).

In addition, in the RPM-grown cells, vimentin was accumulated in the outer cellular membrane and gathered in a dense ring around the nucleus, while a rather even distribution of vimentin could be seen in 1 *g* control cells (Fig. 3). After incubating the cells for 2 h on the RPM, stronger staining was noted compared with the results measured after a 30-min incubation on the RPM (Fig. 3), while disruptions in the network of the outer cellular membrane by the cells examined for vimentin were detected (Fig. 3). These disruptions, extending over time, were detected best after a 4-h incubation on the RPM (Fig. 3). At this time, the accumulation of beta-tubulin around the nucleus also reached its maximum (Fig. 2). After 16 h on the RPM the density of beta-tubulin around the nucleus was lower (Fig. 2) and the immunofluorescence of vimentin was similar to that of the control cells (Fig. 3). After incubation on the RPM for time frames ranging from 30 min to 16 h, the cytoplasmic architecture was damaged. However, after a 24-h incubation on the RPM no morphological changes were detected in either microtubules (beta-tubulin) or intermediate filaments (vimentin) of the investigated human chondrocytes (Fig. 2–3).

**Figure 4:**
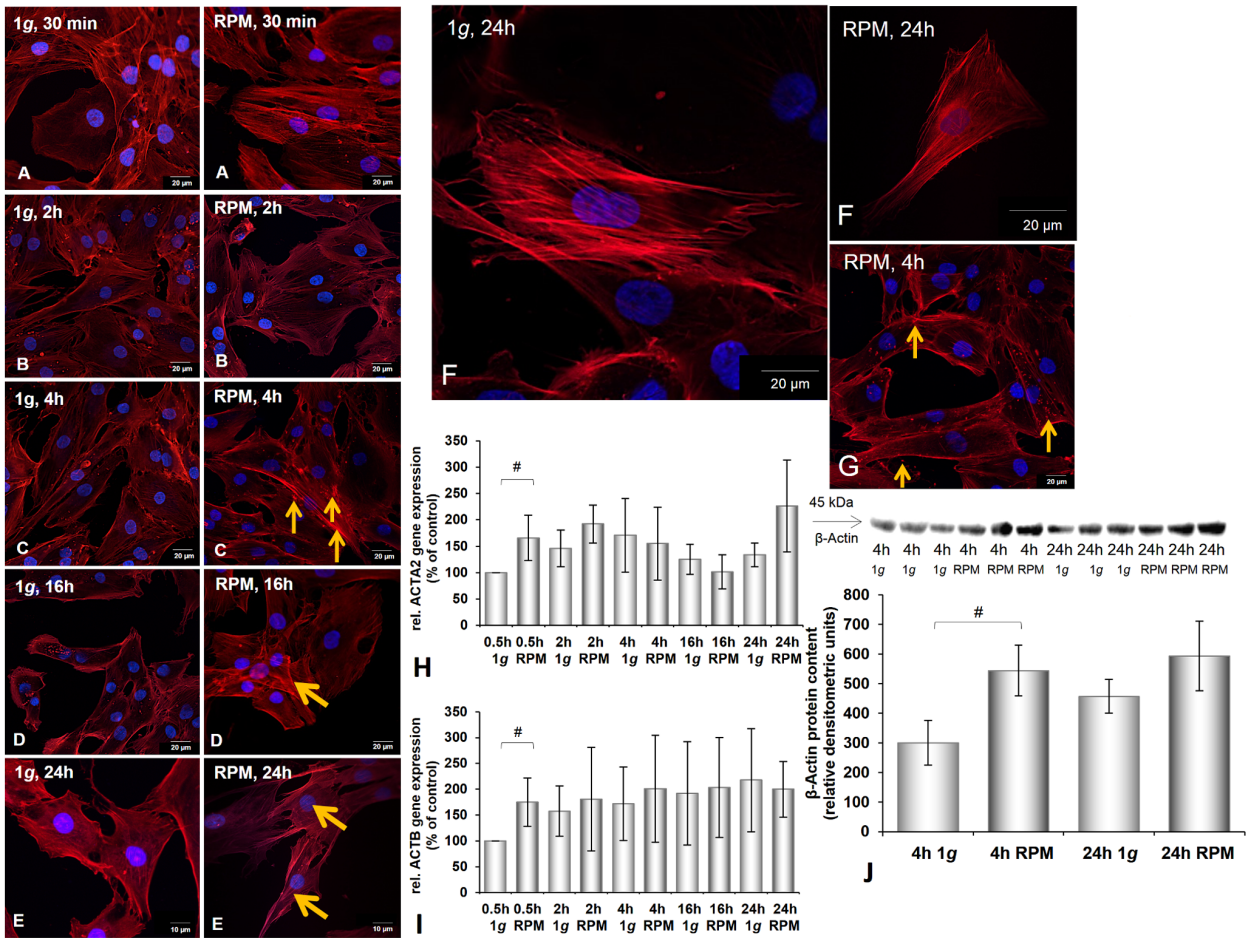
Integrity of nuclei counter-stained by Hoechst 33342, distribution of F-Actin, *ACTA2* and *ACTB* gene expression, and Western blot analysis of beta-actin of chondrocytes cultured at 1 *g* or on the RPM. **A–E:** Integrity of nuclei counter-stained by Hoechst 33342 and distribution of F-Actin in human chondrocytes cultured at 1 *g* or on the RPM for different times (30 min (**A**), 2 h (**B**), 4 h (**C**), 16 h (**D**), 24 h (**E**)). After 30 min and 2 h stress fibres were visible in RPM samples (**A, B**). After 4 h accumulations of F-actin were visible in the outer cellular membrane (**C, G**). The cells were smaller after 24 h RPM exposure (**F**). **H, I:** *ACTA2* and *ACTB* gene expression: Early up-regulation of these mRNAs was found after 0.5 h. **J:** Western blot analysis of beta-actin: Beta-actin clearly increased after 4 h incubation on the RPM. Data are given as mean ± standard deviation; #P<0.05; *P<0.005.

The shape of the chondrocytes also changed, as visualised by F-actin staining: Stress fibres were visible after RPM exposure of 4 h (Fig. 4). The fibres became more elongated and thinner during further incubation on the RPM compared to the control cells incubated for up to 24 h under normal gravity (Fig. 4).

**Figure 5:**
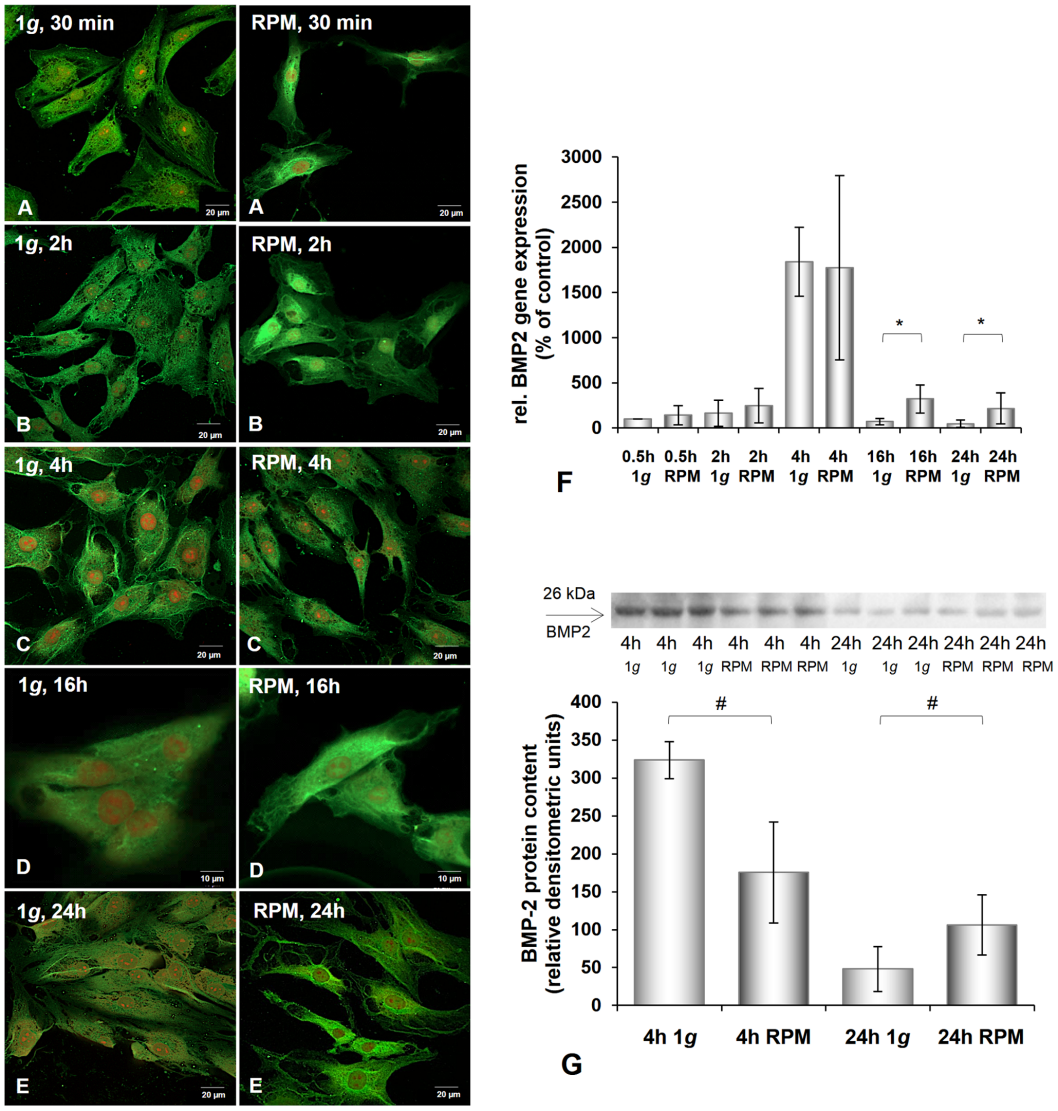
Immunofluorescence, gene expression, and Western blot analysis of BMP-2 of chondrocytes cultured at 1 *g* or on the RPM. **A–E:** BMP-2 immunofluorescence of chondrocytes cultured at 1 *g* or on the RPM for different times (30 min (**A**), 2 h (**B**), 4 h (**C**), 16 h (**D**), 24 h (**E**)). **F:** Gene expression pattern of *BMP2* in chondrocytes after 30 min, 2 h, 4 h, 16 h, and 24 h incubation on the RPM and under static control conditions (1 *g*). The mRNA was up-regulated after 24 h. After 4 h clear up-regulation was detected in 1 *g* and RPM cultures, but this was blunted after 16 h. **G:** Western blot analysis of BMP-2 protein revealed a decrease after 4 h and an increase after 24 h RPM exposure. Data are given as mean ± standard deviation; #P<0.05; *P<0.005.

The staining intensity of the cells treated with antibodies against BMP-2 was higher under simulated microgravity conditions, compared with the corresponding 1 *g* cultures (1 *g*) (Fig. 5).

### Effects of Microgravity on Gene Expression

Quantitative real-time reverse transcriptase PCR of individual gene expression levels (normalised to 18S ribosomal RNA) showed a clear effect of simulated microgravity on all genes examined. To determine the changes in gene expression and statistical dependence, we calculated the means and the standard deviation of the expression of every gene from all six chondrocyte cell lines and compared them with each other. The expression levels of the 1 *g* group after incubation for 30 min was defined as the 100% value.

**Figure 6:**
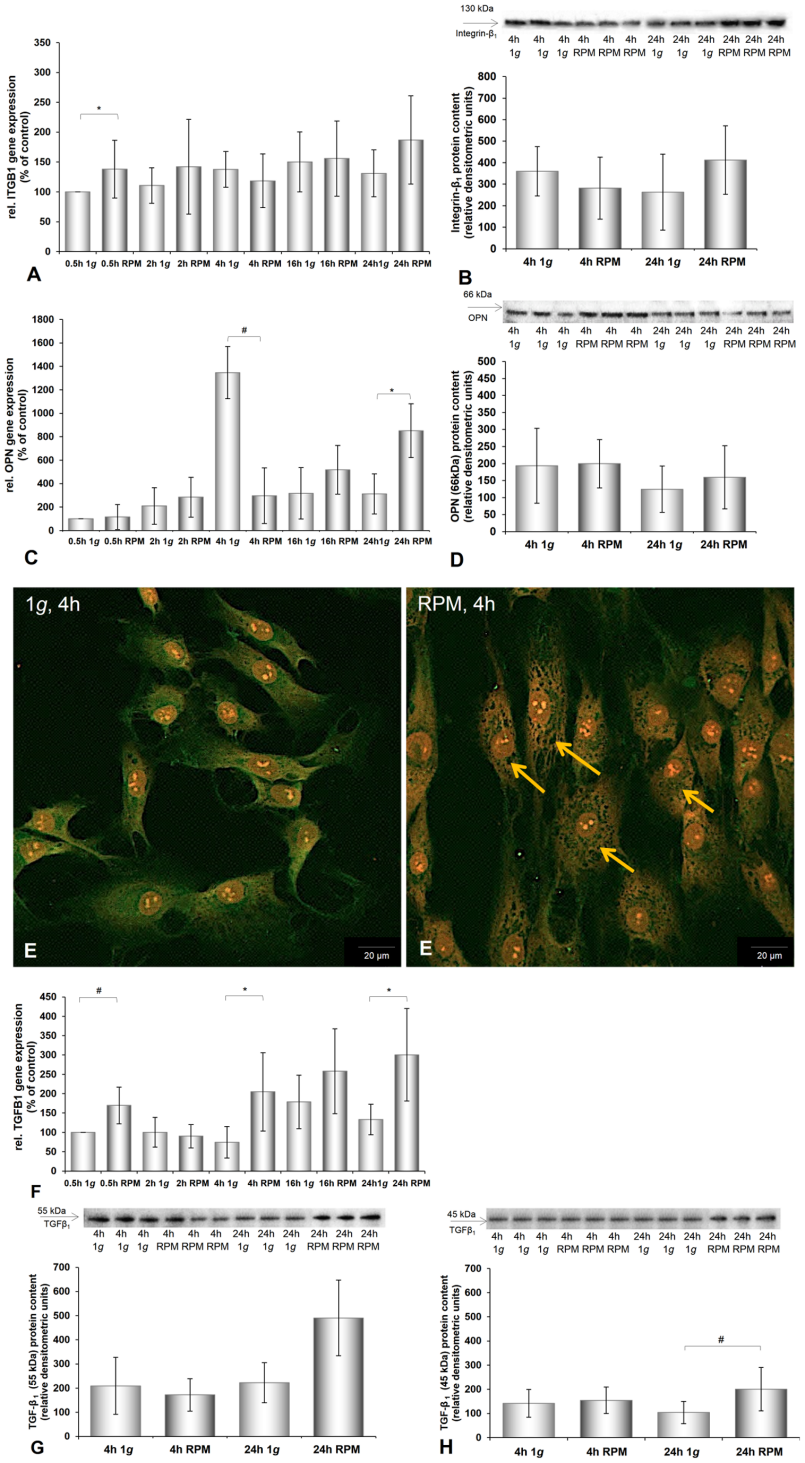
Immunofluorescence of TGF-ß_1_, *ITGB1*, *OPN*, and *TGFB1* gene expression and protein level of chondrocytes cultured at 1 *g* or on the RPM. **A:** The *ITGB1* gene expression and **B:** the integrin-ß_1_ protein level remained the same in both conditions. **C:** The *OPN* mRNA was significantly up-regulated after 24 h on the RPM, whereas the osteopontin protein content was only slightly elevated after 24 h on the RPM (**D**). **E:** Immunofluorescence of TGF-ß_1_ of chondrocytes cultured at 1 *g* or on the RPM. After 4 h culturing on the RPM holes were visible in the cytoskeleton (orange arrows). **F:** *TGFB1* gene expression: The *TGFB1* mRNA was up-regulated after 30 min, 4 h, and 24 h RPM exposure. **G, H:** Western blot analyses of TGF-ß1 protein. The 45-kDa protein was significantly elevated after 24 h RPM exposure (**G**). Data are given as mean ± standard deviation; #P<0.05; *P<0.005.

Besides the BMP2 gene, all investigated genes were already up-regulated after 30 min of culturing on the RPM (Fig. 1, 2, 3, 4, 5). *ACTA2*, *ACTB*, *TUBB6*, *ITGB1*, and *TGFB1* genes were still up-regulated after 2 h, 4 h, 16 h, and 24 h compared with the corresponding 1 *g* group. The *VIM* gene was down-regulated after 4 h incubation on the RPM compared with 1 *g* samples and still, but not significantly, down-regulated after 16 h and 24 h incubation on the RPM (Fig. 3). The *BMP2* and *OPN* genes showed extreme down-regulation after 4 h incubation compared to the controls (Fig. 5, 6). The cells incubated on the RPM for 4 h also revealed up-regulation of the *BMP2* gene, but without any significance, and down-regulation of the *OPN* gene.

### Effects of RPM Exposure on the Production of Extracellular Matrix Proteins

The chondrocytes cultured on the RPM for 4 h showed an increase in beta-tubulin and beta-actin proteins and a reduced amount of BMP-2 (Fig. 2, 4, 5). However, after 24 h of RPM exposure, we detected an elevation of vimentin (Fig. 3), beta-actin (Fig. 4), BMP-2 (Fig. 5), and TGF-β_ 1_ protein (Fig. 6), while the beta-tubulin protein content did not change compared to the corresponding static control cells (Fig. 2).

4 h and 24 h of simulated microgravity using the RPM did not influence the protein levels of integrin-beta_1_ (Fig. 6), osteopontin (Fig. 6), or type 2 collagen (Fig. 1).

## Discussion

This is the first study demonstrating the short-term effects of simulated microgravity on human chondrocytes, cultured on an RPM for 30 min, 2 h, 4 h, 16 h, and 24 h. It is known that long-term microgravity induces a variety of changes such as alterations of the cytoskeleton, and changes in growth, proliferation, differentiation, migration and adhesion in human cells [25], [26]. Changes in growth behaviour have previously been reported for chondrocytes, which form three-dimensional aggregates after several days of cultivation on an RPM [10]. The *in vitro* culture of bovine chondrocytes on synthetic polymer scaffolds was investigated on the MIR Space Station and on Earth [14]. Freed *et al.* [14] first cultured these cells for 3 months in rotating bioreactors on Earth and then for 4 more months on either the MIR or on Earth (1 *g*). Both conditions delivered cartilaginous constructs [14].

The response of any organism to gravity depends ultimately on functions at the cellular level. Real and simulated microgravity induce early alterations of the cytoskeleton in different kinds of human cells, such as thyroid cancer cells [27], [28], endothelial cells [23], and glial cells [29]. Uva *et al.* demonstrated cytoskeletal changes occurring in glial cells (C(6) line) after being cultured for 15 min, 30 min, 1 h, 20 h and 32 h under simulated microgravity [29]. Thus, it is important to study the direct effect of gravity on single cell functions [25], [26].

After as little as 30 min of incubation under simulated microgravity, human chondrocytes seem to switch to a path guiding them to change their morphology and rearrange their cytoskeletal proteins, alter their gene expression and eventually initiate a three-dimensional cell–cell aggregation. The cells used in this study were freshly digested, seeded directly into flasks and used for the RPM experiment or as 1 *g* controls after they reached sub-confluency in low passages (P2 and P3). This is important, as during serial passages, chondrocytes can de-differentiate into cells, presenting a fibroblast-like phenotype and producing predominately type I collagen. It is well known that during *in vitro* culture of chondrocytes, the phenotype of these cells is unstable and rapidly lost during passaging in monolayer cultures [30]. This process is termed de-differentiation and is characterized by the loss of cellular ability to synthesise type II collagen [30]. Hence, it is important to note that the chondrocytes used for the study permanently produced a high amount of collagen type 2 and did not change their morphology before the RPM experiments (Fig. 1).

The chondrocyte cytoskeleton comprises actin microfilaments, tubulin microtubules and vimentin intermediate filaments [31]. The F-actin cytoskeleton has been implicated in changes in cell shape and function as well as signaling processes under microgravity. Studies performed on sounding rockets revealed that the amount of F-actin increased in A431 epidermoid carcinoma cells after 7 min under real microgravity [32], leading to the suggestion that the actin microfilament system is sensitive to changes in gravity and that remodeling of actin microfilaments may affect signal transduction [32]. We have previously investigated thyroid cancer cells during a parabolic flight mission and detected early alterations in the actin cytoskeleton. After 22 s microgravity, F-actin was altered, and the *ACTB* mRNA was significantly up-regulated after the first and thirty-first parabolas [33].

The first cytoskeletal changes in the morphology of human chondrocytes were observed after 30 min incubation on the RPM. The most remarkable changes were detectable after 4 h. At this time point, the perinuclear accumulation of beta-tubulin in the microtubules, crucial to many fundamental processes including cell motility and division [34], reached its maximum, and disruptions in the vimentin microfilament, important for signal transduction processes [34], were observed. After 24 h incubation of human chondrocytes on the RPM we were unable to detect any visible changes in the cytoskeletal morphology compared to control cells. This indicates the capacity of chondrocytes to adapt to a stressful environment. Adaptation phenomena have previously been seen in glial cells after 32 h incubation under simulated microgravity [29]. However, the chondrocytes were still viable (Fig. 1, Fig. 4) and showed decreased deposition of acidic polysaccharides (Alcian Blue staining). This finding was also observed in chondrocytes cultured on the ISS during a 10-day flight [14]. Moreover, after 24 h of culturing on the RPM the cells changed their shape, becoming more elongated.

In addition, we detected changes in the gene expression levels after as little as 30 min incubation on the RPM. *ACTA2, ACTB, VIM, TUBB6, ITGB1* and *TGFB1* mRNAs were significantly up-regulated after 30 min RPM exposure as measured by quantitative rtPCR. These results are consistent with the observed early changes in the cytoskeleton of human chondrocytes cultured on the RPM. Interestingly, BMP-2 and osteopontin mRNA showed extremely high expression levels in the control samples incubated under normal gravity conditions for 4 h. Moreover, the protein content of BMP-2 after incubation for 4 h under simulated microgravity was high. To investigate the cause of this up-regulation further experiments are required. However, BMP-2, a member of the TGF- β superfamily, has been proposed as a tool for cartilage repair and as a stimulant of chondrogenesis, which rarely occurs in healthy cartilage [35]–[37].

After a 24 h incubation of human chondrocytes on the RPM, we detected up-regulation of TGF-β_1_ gene expression as well as elevated production of TGF-β_1_ protein. TGF-β_1_ is known to promote the disruption of follicles, cell spreading, migration, and confluence in porcine thyroid cells by a mechanism that does not involve cell proliferation [38]. It also induces the formation of a tight monolayer and domes [38]. TGF-β_1_ enhances the production of various structural proteins [38], [39]. Thus, it is tempting to assume that this protein plays a role in microgravity-induced enhancement of ECM production in chondrocyte cells, as in endothelial cells [40] and thyroid cancer cells [27]. Thyroid cancer cells displayed an increase in TGF-ß_1_ protein when cultured on the RPM [27]. In addition, human endothelial cells exhibited enhanced TGF-β_1_ gene expression after 4 d and significantly enhanced ECM proteins after 48 h [13], [18].

The RPM system is not equivalent to real microgravity. Tissues cultures on the RPM generally displayed intermediate characteristics compared with ISS and 1 *g* conditions [15]. The RPM is a suitable device for tissue engineering of several tissues such as three-dimensional spheroids, cartilage or intima constructs [10], [11], [15], [18], [19], [23], [41]. Nevertheless, the RPM- and ISS-induced morphological features were similar. We have previously demonstrated that human follicular thyroid cancer cells, when cultured in Space, grow in the form of extraordinary large three-dimensional aggregates with altered expression of EGF and CTGF genes [42]. Therefore, a direct comparison is important to confirm whether cells exposed to simulated microgravity respond in a similar way to real microgravity conditions.
